# Obesity in Inflammatory Bowel Disease: Gains in Adiposity despite High Prevalence of Myopenia and Osteopenia

**DOI:** 10.3390/nu10091192

**Published:** 2018-09-01

**Authors:** Robert Venning Bryant, Christopher G. Schultz, Soong Ooi, Charlotte Goess, Samuel Paul Costello, Andrew D. Vincent, Scott N. Schoeman, Amanda Lim, Francis Dylan Bartholomeusz, Simon P.L. Travis, Jane Mary Andrews

**Affiliations:** 1 IBD Service, Department of Gastroenterology and Hepatology, The Queen Elizabeth Hospital, 28 Woodville Road, Woodville 5011, Australia; sam.costello@sa.gov.au (S.P.C.); snschoeman@gmail.com (S.N.S.); Amanda.Lim@sa.gov.au (A.L.); 2 School of Medicine, Faculty of Health Sciences, University of Adelaide, Adelaide 5000, Australia; jane.andrews@sa.gov.au; 3 Department of Nuclear Medicine, PET and Bone Densitometry, Royal Adelaide Hospital, Port Road, Adelaide 5000, Australia; chris.schultz@sa.gov.au (C.G.S.); Dylan.Bartholomeusz@sa.gov.au (F.D.B.); 4 IBD Service, Department of Gastroenterology and Hepatology, Royal Adelaide Hospital, Port Road, Adelaide 5000, Australia; soongyuan.ooi@uq.edu.au (S.O.); charlotte.goess@sa.gov.au (C.G.); 5 Freemasons Foundation Centre for Men’s Health, School of Medicine, University of Adelaide, North Terrace, Adelaide 5000, Australia; andrew.vincent@adelaide.edu.au; 6 Translational Gastroenterology Unit, John Radcliffe Hospital, Headley Way, Headington, Oxford OX3 9DU, UK; simon.travis@ndm.ox.ac.uk

**Keywords:** body composition, obesity, visceral adipose tissue, fat, osteoporosis, osteopenia, sarcopenia, inflammatory bowel disease

## Abstract

Background: Rising rates of obesity have been reported in patients with inflammatory bowel disease (IBD); however, prospective data is lacking. The aim of this study is to prospectively evaluate body composition in adults with IBD over 24 months. Methods: Whole body dual energy X-ray absorptiometry (DXA) data was performed at 0 months, 12 months, and 24 months. Bone mineral density (BMD), fat mass index (FMI (kg)/height (m^2^)), appendicular skeletal muscle index (ASMI (kg)/height (m^2^)), visceral adipose tissue and the visceral adipose height index (VHI, VAT area (cm^3^)/height (m^2^)), and clinical and anthropometric assessments were performed at each time point. Multivariable linear mixed effects regression analyses were performed. Results: Initially, 154 participants were assessed at baseline (70% Crohn’s disease, 55% male, median age 31 years), of whom 129 underwent repeated DXA at 12 months, and 110 underwent repeated DXA at 24 months. Amongst those undergoing repeated DXA, their body mass index (BMI) significantly increased over time, such that by 24 months, 62% of patients were overweight or obese (annual change BMI β = 0.43, 95%CI = [0.18, 0.67], *p* = 0.0006). Gains in BMI related to increases in both FMI and VHI (β = 0.33, 95%CI = [0.14, 0.53], *p* = 0.0007; β = 0.08, 95%CI = [0.02, 0.13], *p* = 0.001; respectively), whereas ASMI decreased (β = −0.07, 95%CI = [−0.12, −0.01], *p* = 0.01) with a concordant rise in rates of myopenia (OR = 3.1 95%CI = [1.2, 7.7]; *p* = 0.01). Rates of osteopenia and osteoporosis were high (37%), but remained unchanged over time (*p* = 0.23). Conclusion: Increasing rates of obesity in patients with IBD coincide with decreases in lean muscle mass over time, while high rates of osteopenia remain stable. These previously undocumented issues warrant attention in routine care to prevent avoidable morbidity.

## 1. Introduction

Body composition refers to proportions of bone, fat, and fat-free (lean) mass in the body and may be abnormal in many patients with inflammatory bowel disease (IBD) [1]. Despite the potential negative effects of disturbances in body composition on IBD-related outcomes, response to therapy, cardiovascular disease, and quality of life (QoL), there is a paucity of prospective data on body composition in patients with IBD [1,2].

Rates of obesity are rising in patients with IBD, as in the general population; 15–40% of adults with IBD are obese, and 20–40% are overweight [3]. IBD may be an independent risk factor for obesity that is driven by dysbiosis and aberrations in intestinal microbial metabolism [4,5]. Treatments used for IBD, in particular corticosteroids and anti-tumor necrosis factor alpha therapies, may also play a role in obesity [3]. On the other hand, adipose tissue is metabolically active, and could plausibly contribute to a pro-inflammatory susceptibility to IBD [4,6]. However, existing data on the impact of obesity on IBD susceptibility and disease course are conflicting. This is partly due to the use of the body mass index (BMI) as a blunt instrument for measuring adiposity, which is unable to distinguish between adiposity compartments with distinct metabolic profiles [3,6,7,8,9,10]. Visceral adipose tissue (VAT) is increased in many patients with Crohn’s disease (CD) and has been associated with a complicated CD phenotype [3,4,6,11,12,13,14]. There are no longitudinal prospective data on direct measures of adiposity in patients with IBD.

There are emerging reports of high rates of both myopenia, which is defined as low lean mass, and sarcopenia, which is defined as a low lean mass coupled with loss of strength, in patients with IBD [1,15,16,17]. This matters, because low lean mass has been associated with an increased need for surgery and poor surgical outcomes in IBD, as well as with osteopenia [18,19,20]. Lean mass deficits may be difficult to detect in clinical practice, where BMI can be falsely reassuring [17,18]. Current data on sarcopenia in IBD are limited by small sample sizes and retrospective study designs, as well as a lack of an appropriate incorporation of a functional assessment [1]. 

A deficit in bone mineral density (BMD) (osteoporosis/osteopenia) is one of the most common complications of IBD, and has been reported in 20% to 50% of patients [21,22,23]. Reduced BMD is associated with an increased risk of pathological fractures and associated morbidity [22,24]. The pathogenesis of metabolic bone disease in IBD is likely multifactorial; however, clinical data are lacking, and the influence of IBD-related factors beyond conventional risk factors remains poorly explored [25,26].

Dual-energy X-ray absorptiometry (DXA) is in routine use in patients with IBD for the measurement of BMD. Whole body DXA adds little in terms of time and radiation, yet gleans accurate and regional body composition information and is considered a gold-standard tool for the evaluation of body composition as compared to cross-sectional imaging [1,27].

Therefore, the aims of this study were:

1. To evaluate body composition in patients with IBD, with serial prospective measurements over time;
2. To explore the influence of clinical factors on body composition in patients with IBD;
3. To explore whether standard anthropometric testing can detect aberrations in body composition.

## 2. Materials and Methods

### 2.1. Subjects

**Figure 1:**
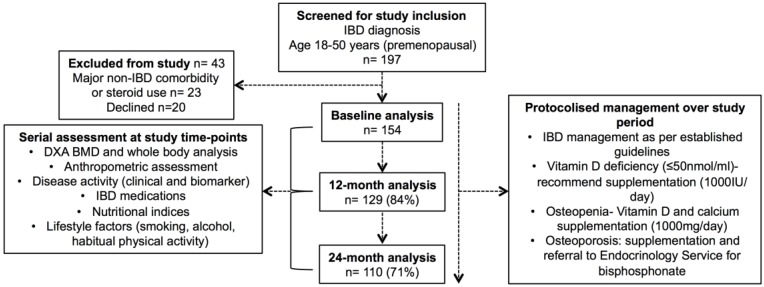
CONSORT diagram—inflammatory bowel disease (IBD) cohort. Legend: DXA, dual energy X-ray absorptiometry, BMD, bone mineral density.

Consecutive patients with IBD (aged 18–50 years and pre-menopausal if female) managed by a tertiary IBD service were invited to participate in a prospective study between April 2012 and September 2013 (Figure 1). Those with significant medical or surgical comorbidity other than IBD, current pregnancy, or steroid use other than that required for IBD, were excluded. 

### 2.2. Subject Data Collection

Prospective data were captured at 0 months, 12 months, and 24 months. At each study time-point case note, an IBD database and medical prescription review was undertaken. Data capture included demographics, Montreal classification at baseline, current IBD therapy, cumulative lifetime corticosteroid use (equivalent to prednisolone ≥10 mg/day) and IBD-related surgery [28]. IBD disease activity was assessed at each study time point using clinical indices and biomarkers of inflammation (C-reactive protein (CRP) and faecal calprotectin (FC) using a CALPRO^®^ ELISA test). CRP ≥ 5 mg/L and/or FC ≥ 100 μg/g were considered consistent with active disease as a composite biomarker assessment. 

Lifestyle factors were assessed at 0 months, 12 months, and 24 months. Average alcohol consumption was dichotomised into <20 g/day versus ≥20 g/day. Habitual physical activity was assessed using a validated self-administered Short International Physical Activity Questionnaire (IPAQ), which approximates metabolic equivalents MET-minutes per week and allows stratification into low, medium, and high activity groups [29].

### 2.3. Body Composition, Anthropometric Assessment, and Nutritional Assessment

Dual energy X-ray absorptiometry (DXA, General Electric Lunar Prodigy Vision bone densitometer (system DF + 13727; Encore version 13.60, Madison WI, USA)) of the lumbar spine, total femur, and whole body was used to evaluate BMD and body composition at 0 months, 12 months, and 24 months using standard protocols [30].

Standard calculations of fat and muscle body components were made from DXA data. Appendicular skeletal muscle (ASM) mass, which is considered functionally relevant lean mass, was calculated as the sum of the lean mass of the arms and legs (kg) [31]. The ASM index (ASMI) is the ASM mass in kg divided by the height in metres, squared [32]. Fat mass (FM) is the sum of the body fat mass (kg). The fat mass index (FMI) is calculated as FM in kg divided by height in metres, squared [32]. VAT was measured using CoreScan^®^ analysis software, which correlates well with CT scan measurements [33]. VAT was measured in terms of volume (cm^3^), mass (grams), visceral adipose tissue/height index (VHI, VAT volume (cm^3^)/height in metres, squared), and VAT: subcutaneous adipose tissue (SAT) ratio. Height (m), weight (kg), waist circumference (cm), and hip circumference (cm) were measured at the time of the DXA and the waist–hip ratio (WHR) was calculated.

World Health Organisation (WHO) standard categories for BMI were used: <18.5 kg/m^2^ (underweight), 18.5–24.9 kg/m^2^ (normal weight), 25–29.9 kg/m^2^ (overweight), ≥30 kg/m^2^ (obese) [34]. Data from the Australian Bureau of Statistics National Health Survey 2014–2015 provided numerical comparisons with age and sex-matched Australian population for BMI and waist circumference [35]. Population-based, age and gender-matched normative data and standard deviation (SD) values from the National Health and Nutritional Examination Survey (NHANES) were used to calculate the *z*-scores for ASMI and the fat mass index (FMI) [17,32]. WHO population-based, age and sex-matched normative data of the lumbar spine and femur BMD were used to calculate BMD *t*-scores and *z*-scores [36]. The lowest BMD *t*-score at either site was used to stratify patients as normal (>−1), osteopenic (≤−1 >−2.5), or osteoporotic (≤−2.5).

Isometric handgrip strength was measured using a Jamar^®^ Digital Hand Dynamometer, representing a robust surrogate measure of whole body strength [31]. Grip strength *z*-scores were calculated from population-based age and sex-matched normative data derived from healthy adult controls [17]. 

Myopenia was defined as an ASMI ≥1 SD below the age and gender-matched ASMI mean [31]. Sarcopenia was defined using combined anatomical and functional criteria, as both ASMI and grip strength >1 SD below the age and gender-matched means [31].

Nutritional assessment was performed at each study time point using clinical and laboratory criteria, including albumin, vitamin D (automated chemiluminescent assay), calcium, haemoglobin, iron studies, vitamin B12, and folate. Low vitamin D was defined as serum 25(OH) vitamin D level <50 nmol/L.

### 2.4. Management during Prospective Study Period

The routine IBD care of enrolled subjects within the tertiary IBD service was consistent with international guidelines (Figure 1) [37,38]. The protocolised management of bone health and nutrition was undertaken during the study period. Oral vitamin D supplementation was recommended to those with low levels (<50 nmol/L). All of the patients were advised to follow a calcium-rich diet and engage in regular exercise. Patients who were found to be osteopenic (or at risk of osteopenia with current corticosteroid use) were advised to take calcium supplementation (1000 mg/day) and oral vitamin D supplementation (≥1000 IU/day). Patients found to be osteoporotic were referred to the Endocrinology service for consideration of bisphosphonate therapy.

### 2.5. Ethical Considerations

The study was approved by the Royal Adelaide Hospital Research Ethics Committee (#120304). Whole-body DXA does not confer significant additional radiation to standard BMD assessment. Radiation safety reported that the total radiation dose per DXA study visit was 2.56 μSv.

### 2.6. Statistical Methods

Continuous outcomes are presented using means, standard deviations, medians, and interquartile ranges (IQR), with categorical outcomes as counts and percentages, unless otherwise stated. The lack of existing prospective data on body composition in IBD did not allow a formal power calculation. The enrolment of 150 patients was considered sufficient to provide adequate power for multivariable analysis, allowing for a dropout rate of 30% over the 24 months. 

***Change over time.*** Changes in disease activity, treatment, nutrition, and anthropometric and body composition variables in individual patients who underwent repeated measures testing over the 24-month study period were assessed using either linear or logistic mixed effects models, where appropriate. The β coefficient describes the change in the body composition variable per unit of time in the study (12 months). In all of the models, time since baseline assessment (years) was the linear fixed effect, with random intercepts per individual. Compound symmetric and unstructured variance–covariance were assumed for the linear and logistic models respectively. In all of the models, residuals and random effect estimates were examined to ensure that the model distributional assumptions appeared satisfied. As a consequence, VHI was log transformed. Missing data values were not included in the analysis.

***Assessment of factors associated with body composition.*** Linear mixed-effects models were constructed to explore prospective clinical associations with serial assessments of BMI, FMI, VHI, ASMI, and BMD (lumbar spine t-score) at 0 months, 12 months, and 24 months. Univariable models were constructed, followed by full multivariable models. We report β coefficients that describe the change in the dependent variable per unit change in a covariate. Missing data were imputed with cohort means. Significance was set at the 5% alpha level (two-sided). Analyses were performed on R software v3.4.3 (R Studio, Boston, MA 02210, USA) using the nlme and lme4 packages.

## 3. Results

### 3.1. Subject Characteristics

Overall, 197 patients with IBD were assessed for eligibility during the study enrolment period (April 2012–September 2013), 43 of whom were excluded (Figure 1). Initially, 154 patients were enrolled in the study at baseline, of whom 129 (84%) completed the 12-month and 110 (71%) completed the 24-month study assessment. Cross-sectional analysis of the cohort at baseline has been previously published (Supplementary Table S1) [17]. Only those patients who underwent repeated measures (*n* = 129) were included in the primary analysis (Table 1).

In brief, 95 (74%) patients had CD, and 34 (26%) had ulcerative colitis (UC) (Table 1). The median age of the cohort was 31 years (IQR 25–40), with a median IBD disease duration of 7.7 years (IQR 4.5–12.3). The cohort was predominantly Caucasian (*n* = 121, 94%). Active disease, defined by composite biomarker assessment, was present in 65 patients (50%) at baseline. Prior to enrolment, 38/95 (40%) patients with CD and 1/34 (1%) patient with UC had undergone abdominal surgery. At baseline, 53 (41%) of patients were prescribed biologic therapy, the use of which increased over the study period (59 (54%) patients were prescribed biologic therapy at 24 months, *p* = 0.004). Oral corticosteroid therapy was currently prescribed for 38 (29%) patients, which significantly decreased over time (*p* < 0.0001). Vitamin D levels were low in 52 (40%) of the patients at baseline, 50/52 (96%) of whom were prescribed replacement therapy. Accordingly, there was a significant increase in vitamin D levels over the study period (*p* = 0.01). 

### 3.2. Baseline Body Composition

A detailed cross-sectional analysis of the body composition of this cohort at baseline (*n* = 137, as enrolled until July 2013) has been previously published [17].

In brief, of those who underwent repeated measures testing (*n* = 129), the mean BMI at baseline was 26.5 ± 5.1; with 33 (26%) patients classified as overweight and 30 (23%) patients classified as obese (Table 2). Myopenia was evident in 24 (19%) patients, and functional sarcopenia was evident in 12 (9%) patients. Low BMD was prevalent at baseline; with 45 (35%) patients classified as osteopenic and 3 (2%) patients classified as osteoporotic (48 (37%), with either osteopenia or osteoporosis).

### 3.3. Changes in Body Composition over 24 Months

**Figure 2:**
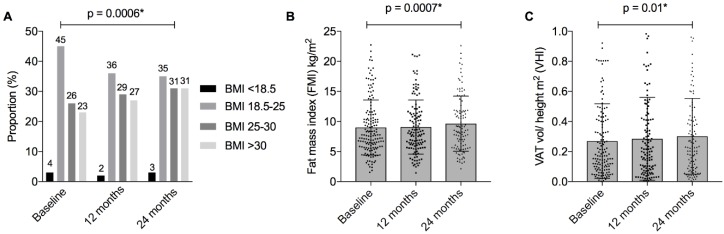
BMI and measures of adiposity in patients with IBD over 24 months. **Figure Legend:** (**A**) Body mass index (BMI) categories according to World Health Organisation criteria; (**B**) Fat mass index (kg/height m^2^). Mean and standard deviation presented; (**C**) Visceral adipose tissue (VAT) height index (VAT volume cm^3^ /height m^2^). Mean and standard deviation presented. * *p* < 0.05, denoting statistical significance.

***Anthropometrics.*** BMI increased over the study period (annual change β = 0.43, 95%CI = [0.18, 0.67], *p* = 0.0006), as did the proportion of patients categorised as overweight and obese (at 24 months 31% overweight and 31% obese) (Table 2, Figure 2). Waist circumference increased over time (β = 1.4, 95%CI = [0.4, 2.3], *p* = 0.003), although no significant change in WHR was observed. Mean BMI, waist circumference, and the proportions of people characterised as overweight or obese, were each numerically higher in the IBD cohort compared to the Australian population stratified by age and gender (Supplementary Table S2).

**Figure 3:**
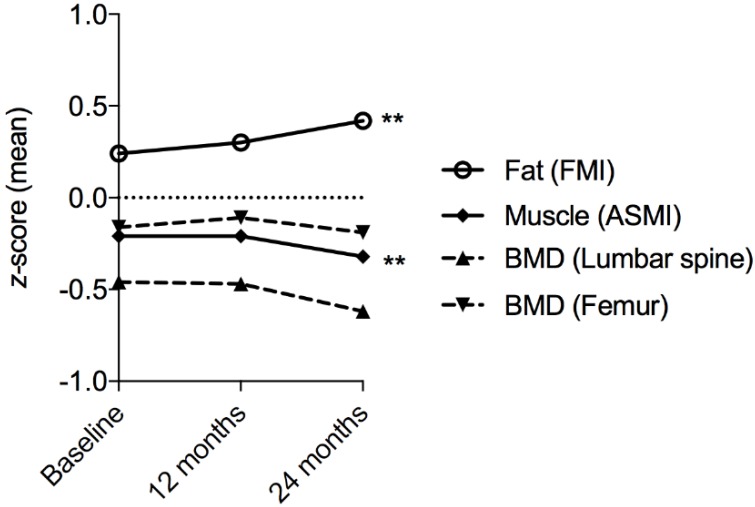
Changes in bone, muscle, and fat in patients with IBD over 24 months. **Figure Legend:** Summary of mean *z*-scores for fat mass index (FMI), appendicular skeletal muscle mass index (ASMI), and bone mineral density (BMD), at the femur and lumbar spine. ** *p* < 0.01, denoting statistical significance.

***Adiposity.*** FMI increased significantly over the study period (β = 0.33, 95%CI = [0.14, 0.53], *p* = 0.0007), with a concordant increase in FMI *z*-score (β = 0.08, 95%CI = [0.02, 0.14], *p* = 0.006). (Table 2, Figure 2 and Figure 3) Similarly, VAT volume increased (β = 0.08, 95%CI = [0.02, 0.14], *p* = 0.01), which resulted in an increase in VHI (β = 0.08, 95%CI = [0.02, 0.13], *p* = 0.01), but not VAT:SAT (β = 0.027, 95%CI = [−0.032, 0.087], *p* = 0.36) (Table 2).

**Figure 4:**
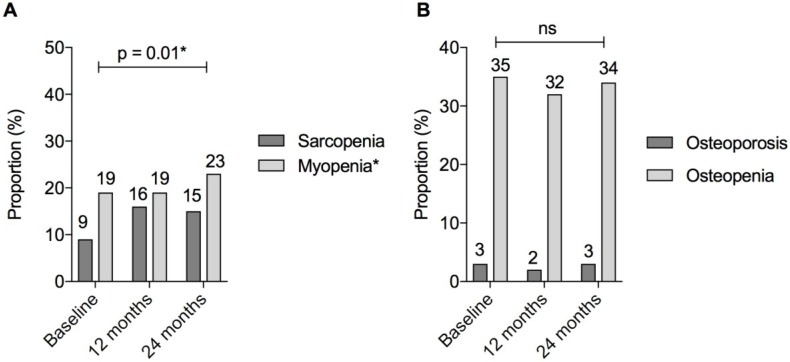
Myopenia, sarcopenia, and metabolic bone disease in patients with IBD over 24 months. **Figure Legend:** (**A**) Sarcopenia defined as BOTH appendicular skeletal muscle index (ASMI) and grip strength <1 standard deviation below gender and age-matched mean. Myopenia defined as appendicular skeletal muscle index (ASMI) <1 standard deviation below gender and age-matched mean; (**B**) osteopenia and osteoporosis according to World Health Organisation criteria. Osteopenia defined as bone mineral density (BMD) 1–2.5 standard deviations and osteoporosis as ≥2.5 standard deviations below the young adult mean. * *p* < 0.05, denoting statistical significance; ns, non-significant *p* value > 0.05.

***Muscle.*** ASMI decreased significantly over the study period (ASMI β = −0.07, 95%CI = [−0.12, −0.01], *p* = 0.01; ASMI *z*-score β = −0.07, 95%CI = [−0.11, −0.02], *p* = 0.002) (Table 2). There was a significant increase in myopenia over the study period (19%, 19%, and 24% at 0 months, 12 months, and 24 months, respectively; OR = 3.1 95%CI = [1.2, 7.7]; *p* = 0.01) (Figure 4). There was a trend towards an increase in the proportion of patients classified as sarcopenic; however, this did not reach statistical significance (OR = 2.4, 95%CI = [1.0, 6.0]; *p* = 0.05).

***Bone.*** Femur BMD *t*-score increased significantly over the study period, but there was no change in lumbar spine BMD *t*-score (β = 0.041, 95%CI = [0.016, 0.066], *p* = 0.001; β = 0.013, 95%CI = [−0.022, 0.048], *p* = 0.47, respectively). No difference in BMD *z*-scores at either site was detected over time (Table 2). Overall, there was no significant change in the proportion of patients classified with osteopenia 37/110 (34%) or osteoporosis 3/110 (3%) (*p* = 0.23), despite the proactive management of bone health over the study period (Figure 4).

### 3.4. Clinical Associations with Serial BMI Measurements

BMI was positively associated with vitamin D levels (β = 0.006, 95%CI = [0.00, 0.0.11], *p* = 0.03). FMI, ASMI, and grip strength were also positively associated with BMI over time (β = 1.0, 95%CI = [1.0, 1.1], *p* < 0.0001; β = 1.3, 95%CI = [1.1, 1.5], *p* < 0.0001; β = 0.028, 95%CI = [0.008, 0.049], *p* = 0.006, respectively) (Table 3).

### 3.5. Clinical Associations with Serial FM and VAT Measurements

Older age was positively associated with VHI (β = 0.040, 95%CI = [0.023, 0.057], *p* < 0.0001) (Supplementary Tables S3 and S4). Male gender was positively associated with VHI, but negatively associated with FMI (β = 0.67, 95%CI = [0.39, 0.95], *p* < 0.0001; β = −2.2, 95%CI = [−2.8, −1.6], *p* < 0.0001, respectively). Serum vitamin D was negatively associated with FMI (β = −0.007, 95%CI = [−0.013, −0.001], *p* = 0.02, *p* = 0.02).

Anthropometric measures (BMI and waist circumference) were associated with a higher FMI (β = 0.52, 95%CI = [0.45, 0.59], *p* < 0.0001; β = 0.07, 95%CI = [0.04, 0.10], *p* < 0.0001, respectively) and VHI (β = 0.08, 95%CI = [0.06, 0.11], *p* < 0.0001; β = 0.015, 95%CI = [0.005, 0.026], *p* = 0.003 respectively). In contrast, grip strength was negatively associated with FMI (β = −0.044, 95%CI = [−0.066, −0.021], *p* < 0.0001).

### 3.6. Clinical Associations with Serial ASMI Measurements

Male gender was associated with a higher ASMI (β = 1.2, 95%CI = [0.9, 1.4], *p* < 0.0001) (Supplementary Table S5). FC was negatively associated with ASMI (β = −0.00043, 95%CI = [−0.00067, −0.00018], *p* = 0.0004), whereas anthropometric measures, BMI, and grip strength were positively associated with ASMI (β = 0.13, 95%CI = [0.11, 0.15], *p* < 0.0001; β = 0.020, 95%CI = [0.011, 0.028], *p* < 0.0001, respectively).

### 3.7. Clinical Associations with Serial BMD Measurements

IBD disease duration was negatively associated with lumbar spine *t*-score (β = 0.0031, 95%CI = [−0.0057, −0.0005], *p* = 0.01), as was grip strength (β = 0.009, 95%CI = [0.002, 0.017], *p* = 0.01) (Supplementary Table S6). Both biologic therapy and corticosteroids (cumulative use) were associated with increase lumbar spine *t*-scores (β = 0.08, 95%CI = [0.01, 0.16], *p* = 0.02; β = 0.10, 95%CI = [0.01, 0.18], *p* = 0.02; respectively). *Neither IBD phenotype nor vitamin D level was associated with changes in the* lumbar spine *t*-score.

## 4. Discussion

This prospective study has demonstrated persistent and progressive disturbances in body composition in people with IBD over a relatively short time frame. The most striking finding was a significant increase in the proportion of patients classified as overweight and obese, driven by gains in adiposity. Conversely, muscle mass decreased, with a concordant rise in rates of myopenia. Despite the proactive management of bone health, rates of osteopenia remained high and unchanged. Abnormal body composition may go frequently unrecognised in clinical practice using BMI alone, leaving patients with IBD at risk of potentially avoidable morbidity.

After 24 months of follow-up, 62% of patients with IBD were overweight or obese despite a median age of just 33 years, which is numerically higher than age and gender- matched Australian population data [35]. Earlier studies of obesity in IBD have failed to take into account direct measures of adiposity [3,10]. This study demonstrates that gains in BMI were associated with significant increases in both overall FM and VAT. VAT is a strong independent predictor of incident cardiovascular disease after adjustment for clinical risk factors, including BMI [39,40]. Similar to to other chronic inflammatory conditions, there is emerging evidence to suggest that patients with IBD are at increased risk of cardiovascular disease, which is now recognised as a prime cause of morbidity in this young demographic [2,41].

How can a link between IBD and obesity be explained? In contrast to retrospective reports, we found no association between measures of adiposity and IBD phenotype, inflammatory burden, or medications over time [3,6,7,8,9,10]. The lack of correlation between obesity and IBD therapy, in particular cumulative corticosteroid use is surprising, yet may be in part accounted for by the significant decrease in rates of corticosteroid use over the study period. Interestingly, serum vitamin D levels were negatively associated with FMI, which may be related to the sequestration of vitamin D in adipose tissue, coupled with lifestyle factors associated with obesity (less outdoor activity) [42]. The majority of patients were classified as either inactive or minimally active, which is likely reflective of the burden of chronic illness in this cohort, yet this was not shown to be associated with BMI. It is possible that dysbiosis in IBD, which is characterised by reduced microbial diversity and an altered microbial metabolic profile, is a predisposing factor to obesity [43,44,45], but this is speculation despite gut metabolomic associations with post-prandial glucose [43,44,45,46].

While adiposity increased, muscle mass (ASMI) decreased. FC, as a measure of luminal inflammation in IBD, was negatively associated with ASMI, which is consistent with the known catabolic effects of chronic inflammation [18,20,47]. Lean mass is important in patients with IBD, and has been shown to have a bearing on response to therapy, surgical outcomes, and quality of life [15,18,20,48,49]. Grip strength proved to be a simple anthropometric test that was independently and positively associated with ASMI, while also negatively associated with FMI.

*IBD disease duration was shown to be negatively associated with BMD; however,* inflammation was not associated with BMD over time, which is consistent with existing data showing a minimal impact of IBD-related inflammation on longitudinal bone loss [21,23,25,26,50]. Likewise, vitamin D levels were not associated with BMD, which is similar to other studies showing an uncertain benefit of vitamin D supplementation on BMD in IBD patients [21,23,25,26,50]. Biologic therapy was found to be positively associated with BMD over time, which is in line with current data [50,51]. The positive association between corticosteroid use and BMD is surprising, and may be a spurious result due to multiple comparisons. *Nevertheless, rates of osteopenia remained high in this cohort, despite not only protocolised management of bone health, but also a warm climate and readily accessible dairy products.*

The limitations of this study are the relatively small sample size, which may have limited statistical power. The study was neither structured nor powered to evaluate the influence of body composition on hospitalisation or surgery over time, nor could it evaluate the impact of these factors on body composition. It did not account for dietary intake, nor were other factors associated with cardiometabolic risk measured (cholesterol, family history, blood pressure). Although a control group was not included, the findings were compared to population-based data [35]. The predominantly Caucasian cohort, which was derived from a single tertiary IBD referral centre, may also limit the generalisability of the findings. On the other hand, it is the first prospective study that has incorporated serial measures of body composition in IBD. Furthermore, DXA, in routine use for measuring BMD, is an affordable tool for monitoring body composition, adding little to the time or radiation of the test [21,33].

This study illustrates the rising rates of obesity in patents with IBD over time, driven by gains in fat mass, while lean mass decreases and metabolic bone disease remains unchanged. Apart from raising concerns about the cardiometabolic risk profile of patients with IBD, it is important that clinicians recognise that an increase in BMI may obscure a decrease in muscle mass (myopenia). The study provides a rationale for measuring body composition, as well as a means to do so with DXA, and supports recommendations for the use of grip strength as a discriminatory anthropometric test in clinical practice.

## Figures and Tables

**Table 1:** Clinical and Nutritional Characteristics of IBD Cohort over 24 Months.

			Baseline	12 Months	24 Months	Δ *p* Value
Demographics		Patients (*n*, %)	129 (84%)	129 (84%)	110 (71%)	-
		Male (*n*, %)	75 (58%)	75 (58%)	62 (58%)	-
		Age (years) (median, IQR)	31 (25–40)	32 (26–41)	33 (27–42)	-
IBD phenotype		Crohn’s disease (*n*, %)	95 (74%)	92 (71%)	79 (72%)	
		Ulcerative colitis (*n*, %)	34 (26%)	37 (29%)	28 (25%)	
		IBD-related abdominal surgery (*n*, %)	39 (30%)	7 (5%)	12 (11%)	-
		IBD-related hospitalisation (*n*, %)	-	22 (17%)	12 (11%)	-
IBD Disease Activity		IBD clinical disease activity score				-
		Crohn’s Disease CDAI Mean ± SD	90 ± 99	71 ± 67	65 ± 58	
		Median, IQR	60 (26–126)	62 (26–110)	49 (20–90)	
		Ulcerative colitis partial Mayo mean ± SD	1.5 ± 2.2	1.4 ± 2.2	1.5 ± 1.9	
		Median, IQR	0.5 (0–2)	0 (0–2)	0 (0–4)	
		C-reactive protein (mg/L) Mean ± SD				0.40
			8.7 ± 21	5.2 ± 11	5.3 ± 8	
		Median, IQR	1.7 (0.5–7.7)	1.9 (0.5–5.8)	1.6 (0.4–6.3)	
		Faecal calprotectin (μg/g)				0.49
		Mean ± SD Median, IQR	247.5 ± 305	155.9 ± 206	179.4 ± 227	
			84 (20–450)	50 (20–205)	93 (20–245)	
		Composite disease activity assessment ^§^ (*n* active disease, %)				-
			65 (50%)	59 (46%)	57 (52%	
IBD Therapy		Oral corticosteroids ^ Current (*n*, %)	38 (29%)	11 (9%)	7 (6%)	<0.0001 *
		Duration usage (mths) Mean ± SD	28 ± 57			
		Median, IQR	6 (1-24)			
		Biologic therapy overall (*n*, %)	53 (41%)	63 (49%)	59 (54%)	0.0004 *
		Infliximab (*n*, %)	35 (27%)	41 (32%)	35 (32%)	
		Adalimumab (*n*, %)	16 (12%)	19 (15%)	22 (20%)	
		Vedolizumab (*n*, %)	2 (2%)	3 (2%)	2 (2%)	
		5-aminosalicylic acid therapy (*n*, %)	55 (43%)	58 (45%)	53 (48%)	0.18
		Immunomodulator therapy Overall (*n*, %)	73 (57%)	78 (60%)	67 (61%)	0.12
		Azathioprine (*n*, %)	46 (36%)	47 (36%)	41 (37%)	
		Mercaptopurine (*n*, %)	6 (5%)	6 (5%)	5 (5%)	
		Methotrexate (*n*, %)	3 (2%)	5 (3%)	4 (4%)	
		Thiopurine/allopurinol (*n*, %)	18 (14%)	20 (16%)	17 (15%)	
Exercise (IPAQ) ^¶^	Continuous	Mean ± SD	4385 ± 5908	5570 ± 8952	4935 ± 6879	0.36
	Median, IQR		2284 (693–5690)	2106 (862–5745)	2445 (942–5558)	
	Categorical	Inactive	50 (39%)	47 (36%)	37 (34%)	
	Minimally active		30 (23%)	24 (19%)	23 (21%)	
	Active		28 (22%)	41 (32%)	35 (32%)	
Nutritional assessment	Albumin (g/dL)	Mean ± SD	40 ± 4.6	40 ± 3.8	40 ± 3.5	0.64
	Median, IQR		40 (37–43)	40 (37–42)	40 (38–42)	
	Haemoglobin (g/L)	Mean ± SD	141 ± 15	141 ± 14	143 ± 13	0.07
	Median, IQR		141 (132–150)	141 (130–149)	144 (136–152)	
	Ferritin (ng/mL)	Mean ± SD	87 ± 85	93 ± 122	115 ± 186	0.09
	Median, IQR		62 (34–106)	63 (35–104)	71 (42–123)	
	Calcium Mean ± SD		2.35 ± 0.11	2.34 ± 0.1	2.34 ± 0.09	0.16
	Median, IQR		2.35 (2.28–2.42)	2.36 (2.29–2.4)	2.34 (2.30–2.4)	
	Vitamin D (nmol/mL) ^#^	Mean ± SD	64 ± 28	65 ± 25	70 ± 25	0.01 *
	Median, IQR		63 (41–84)	65 (48–80)	67 (53–85)	
	Low Vitamin D level		52 (40%)	40 (31%)	38 (35%)	
	Vitamin D supplementation		50 (39%)	40 (31%)	37 (34%)	
	Bisphosphonate therapy		2 (1%)	4 (3%)	4 (4%)	

Analysis performed using linear and logistic mixed effects regressions. Data presented as mean ± standard deviation (SD), median (interquartile range (IQR)), counts and percentage. CDAI, Crohn’s Disease Activity Index; ^§^ C-reactive protein (CRP) ≥5 mg/L and/or faecal calprotectin (FC) ≥100 μg/g were considered consistent with active disease as a composite biomarker assessment. ^ Cumulative months equivalent to prednisolone ≥10 mg daily. ^¶^ International Physical Activity Questionnaire (Short). ^#^ Low Vitamin D level classified as <50 nmol/L, Vitamin D supplementation (≥1000 IU/day), * significant *p* value < 0.05.

**Table 2:** Body composition in IBD patients over 24 months.

Body Composition			Baseline (*n* = 129)	Year 1 (*n* = 129)	Year 2 (*n* = 110)	*p* Value
**Anthropometric assessment**	Body mass index (BMI)	Mean ± SD (kg/m^2^)	26.5 ± 5.1	27.4 ± 5.8	27.7 ± 5.6	0.0006 *
	Δ ± SD		-	0.52 ± 2.51	0.84 ± 2.66	
	Median (IQR)		25.1 (22.9–29.7)	26.5 (23.2–30.7)	26.8 (23.8–30.8)	
	BMI categorical ^¶^	Underweight < 18.5	4 (3%)	3 (2%)	3 (3%)	0.0006 *
	Normal 18.5–25		58 (45%)	47 (36%)	38 (35%)	
	Overweight 25–30		33 (26%)	38 (29%)	34 (31%)	
	Obese ≥ 30		30 (23%)	35 (27%)	34 (31%)	
	Waist circumference (cm)	Mean ± SD	90.8 ± 15.9	92.4 ± 14.5	94.1 ± 14.4	0.003 *
	Δ ± SD		-	0.73 ± 10.32	2.26 ± 9.18	
	Median (IQR)		88 (80–101)	91 (81–102)	93 (83–103)	
	Waist:hip ratio	Mean ± SD	0.88 ± 0.11	0.88 ± 0.10	0.89 ± 0.10	0.31
	Δ ± SD		-	−0.004 ± 0.11	0.01 ± 0.12	
	Median (IQR)		0.86 (0.81–0.95)	0.87 (0.82–0.92)	0.89 (0.83–0.94)	
	Grip strength (PSI)	Mean ± SD	40.2 ± 12.0	39.5 ± 12.2	39.8 ± 12.4	0.68
	Δ ± SD		-	−0.48 ± 5.56	−0.30 ± 5.01	
	Median (IQR)		37.8 (30.0–50.0)	38.0 (29.0–49.3)	37.4 (31.1–49.1)	
	Grip strength *z*-score	Mean ± SD	−0.63 ± 1.30	−0.9 ± 1.49	−0.67 ± 1.23	0.46
	Δ ± SD		-	−0.27 ± 1.55	−0.01 ± 1.27	
	Median (IQR)		−0.48 (−1.29–0.15)	−0.75 (−1.41–0.12)	−0.6 (−1.32–0.15)	
**Fat mass index (FMI)** **(kg/height m^2^)**	FMI (kg/m^2^)	Mean ± SD	8.83 ± 4.57	9.07 ± 4.50	9.64 ± 4.59	0.0007 *
	Δ ± SD		-	0.26 ± 1.54	0.70 ± 2.05	
	Median (IQR)		7.67 (5.67–10.60)	8.09 (6.07–11.32)	8.61 (5.82–11.81)	
	FMI *z*-score	Mean ± SD	0.24 ± 1.26	0.30 ± 1.26	0.42 ± 1.24	0.006 *
	Δ ± SD		-	0.06 ± 0.48	0.17 ± 0.61	
	Median (IQR)		−0.13 (−0.59–0.93)	0.08 (−0.57–0.89)	0.25 (−0.47–1.15)	
**Visceral adipose tissue (VAT)**	VAT volume (cm^3^)	Mean ± SD	834.2 ± 821.7	903.9 ± 901.6	949.9 ± 826.7	0.01 *
	Δ ± SD		-	67.8 ± 331.8	102.1 ± 376.6	
	Median (IQR)		493.7 (216.9, 1175.9)	600.5 (248.8, 1291)	702.1 (266.6–1354)	
	VAT (grams) ^	Mean ± SD	786.9 ± 775.2	852.76 ± 850.53	896.09 ± 779.94	0.01 *
	Δ ± SD		-	64.0 ± 313.0	96.3 ± 355.3	
	Median (IQR)		465.74 (204.59–1109.38)	566.51 (241.63–1209.85)	662.35 (256.23–1274.44)	
	VAT:SAT ^	Mean ± SD	0.64 ± 0.68	0.64 ± 0.55	0.63 ± 0.49	0.36
	Δ ± SD		-	−0.005 ± 0.50	0.026 ± 0.585	
	Median (IQR)		0.45 (0.24–0.8)	0.46 (0.31–0.83)	0.49 (0.28–0.89)	
	VHI ^	Mean ± SD	0.26 ± 0.25	0.28 ± 0.28	0.3 ± 0.25	0.01 *
	Δ ± SD		-	0.022 ± 0.10	0.127 ± 0.604	
	Median (IQR)		0.15 (0.07–0.35)	0.2 (0.08–0.41)	0.23 (0.1–0.41)	
**Bone mineral density (BMD)**	WHO *t*-score femur	Mean ± SD	−0.18 ± 1.02	−0.1 ± 1.03	−0.13 ± 1.03	0.001 *
	Δ ± SD		-	0.075 ± 0.191	0.081 ±0.266	
	Median (IQR)		−0.21 (−0.96–0.52)	−0.20 (−0.99–0.55)	−0.17 (−0.97–0.69)	
	WHO *t*-score spine	Mean ± SD	−0.35 ± 1.1	−0.29 ± 1.11	−0.38 ± 1.17	0.47
	Δ ± SD		-	0.033 ± 0.34	0.027 ± 0.357	
	Median (IQR)		−0.48 (−1.06–0.4)	−0.37 (−1.05–0.39)	−0.36 (−1.21–0.33)	
	WHO *z*-score femur	Mean ± SD	−0.16 ± 1.06	−0.11 ± 1.03	−0.19 ± 0.93	0.86
	Δ ± SD		-	0.049 ± 0.31	0.007 ± 0.52	
	Median (IQR)		−0.24 (−0.93–0.46)	−0.14 (−0.90–0.51)	−0.12 (−0.91–0.48)	
	WHO *z*-score spine	Mean ± SD	−0.46 ± 1.08	−0.47 ± 1.09	−0.62 ± 1.14	0.51
	Δ ± SD		-	0.004 ± 0.385	−0.025 ± 0.41	
	Median (IQR)		−0.52 (−1.19–0.29)	−0.52 (−1.22–0.23)	−0.58 (−1.4–0.18)	
	Bone status ^#^	Osteopenia (*n*, %)	45 (35%)	41 (32%)	37 (34%)	0.23
	Osteoporosis (*n*, %)		3 (2%)	2 (2%)	3 (3%)	
	Overall osteopenia/porosis (*n*, %)		48 (37%)	43 (34%)	40 (37%)	
**Appendicular skeletal muscle index (ASMI) (kg/height m^2^)**	ASMI (kg/m^2^)	Mean ± SD	7.76 ± 1.40	7.80 ± 1.45	7.68 ± 1.40	0.01 *
	Δ ± SD		-	0.014 ± 0.49	−0.127 ± 0.55	
	Median (IQR)		7.60 (6.57–8.48)	7.84 (6.74–8.62)	7.64 (6.66–8.45)	
	ASMI *z*-score	Mean ± SD	−0.21 ± 0.96	−0.21 ± 0.98	−0.32 ± 0.99	0.002 *
	Δ ± SD		-	−0.006 ± 0.392	−0.0129 ± 0.444	
	Median (IQR)		−0.31 (−0.95–0.17)	−0.3 (−0.88–0.24)	−0.45 (−0.93–0.29)	
	Myopenia (Low ASMI ^§^)		24 (19%)	25 (19%)	25 (23%)	0.01 *
	Functional sarcopenia (Low ASMI AND low grip strength ^§^)		12 (9%)	21 (16%)	17 (15%)	0.05

**Table Legend:** Reported are baseline distributions and change from baseline at 12 months and 24 months. Statistical analysis performed using linear and logistic mixed effects regressions. Data presented as mean ± standard deviation, median (interquartile range), counts, and percentage. SD, standard deviation; ^ Data log-transformed prior to analysis: ^ VAT, visceral adipose tissue; ^ VAT:SAT, visceral adipose tissue: subcutaneous adipose tissue ratio; VHI, visceral adipose tissue area (cm^3^) divided by height (m^2^); ^§^ Low appendicular skeletal muscle index (ASMI) and grip strength defined as ≥1 standard deviation below mean. Δ, mean difference from baseline at Year 1 and Year 2 (±standard deviation). ^#^ BMI categories and bone status according to World Health Organisation criteria; * significant *p* value < 0.05.

**Table 3:** Clinical associations with serial body mass index (BMI) measurements over 24 months.

Variable		Univariable		Full Multivariable Model	
		Est. (95%CI)	*p* Value	Est. (95%CI)	*p* Value
Time	Repeated measures over 24 m	0.43 [0.21, 0.65]	<0.0001	0.08 [−0.08, 0.24]	0.28
Demographics	Age at study entry	0.24 [0.15, 0.33]	<0.0001	0.021 [−0.004, 0.046]	0.08
	Gender (Male vs. female)	0.0 [−2.0, 2.0]	0.99	0.38 [−0.18, 0.95]	0.17
IBD-related factors	IBD phenotype (Ulcerative colitis vs. Crohn’s disease)	−0.7 [−2.9, 1.5]	0.52	−0.04 [−0.46, 0.38]	0.86
	IBD disease duration	0.014 [0.005, 0.022]	0.002	0.0003 [−0.0020, 0.0026]	0.78
	Faecal calprotectin (μg/g)	−0.0015 [−0.0025, 0.004]	0.007	0.00021 [−0.00044, 0.00086]	0.48
	C-reactive protein (mg/L)	−0.011 [−0.027, 0.006]	0.19	0.001 [−0.009, 0.011]	0.85
	Current corticosteroid use	−0.4 [−1.0, 0.2]	0.22	−0.10 [−0.49, 0.29]	0.55
	Biologic therapy	0.25 [−0.31, 0.81]	0.38	0.04 [−0.18, 0.25]	0.69
	Immunomodulator therapy	0.42 [−0.03, 0.88]	0.06	0.11 [−0.02, 0.23]	0.08
Lifestyle and nutritional factors	Smoking status Current vs. never Ex vs. never	0.8 [−1.9, 3.4] 2.0 [−0.4, 4.4]	0.23	−0.24 [−0.75, 0.28] 0.34 [−0.11, 0.78]	0.07
	Excess alcohol intake ^	0.2 [−4.9, 5.3]	0.95	0.8 [−0.1, 1.8]	0.06
	Vitamin D level (nmol/mL)	0.006 [−0.005, 0.017]	0.25	0.006 [0.000, 0.011]	0.03
	Habitual exercise (IPAQ score, continuous) ^§^	0.002 [−0.036, 0.041]	0.91	0.009 [−0.011, 0.029]	0.36
	Albumin (g/dL)	0.06 [−0.02, 0.13]	0.16	0.008 [−0.034, 0.049]	0.76
Body composition factors	Fat mass index (FMI)	1.1 [1.0, 1.2]	<0.0001	1.0 [1.0, 1.1]	<0.0001
	Appendicular skeletal muscle index (ASMI)	2.1 [1.8, 2.5]	<0.0001	1.3 [1.1, 1.5]	<0.0001
	Functional sarcopenia ^¶^	−1.5 [−2.5, −0.4]	0.0006	0.11 [−0.41, 0.62]	0.65
	Grip strength (pounds per square inch, PSI)	0.024 [−0.024, 0.072]	0.32	0.028 [0.008, 0.049]	0.006

^ Data log-transformed prior to analysis: ^ VAT, visceral adipose tissue; ^ VAT:SAT, visceral adipose tissue: subcutaneous adipose tissue ratio; VHI, visceral adipose tissue area (cm^3^) divided by height (m^2^); ^¶^ Low ASMI and grip strength ≥1 standard deviation below mean. ^ Excess alcohol use defined according to the Australian Healthy Drinking Guidelines; ^§^ IPAQ, International Physical Active Questionnaire; FMI, fat mass index (kg/height m^2^); ASMI, appendicular skeletal muscle index (kg/height m^2^); Linear mixed effects regression models with missing data imputed with cohort means.

## Supplementary Materials

The following are available online at http://www.mdpi.com/2072-6643/10/9/1192/s1. Supplementary Table S1: Baseline clinical and nutritional characteristics of IBD Cohort; Supplementary Table S2: Body mass index and waist circumference by age as compared to data from the Australian Bureau of Statistic National Health Survey First Results 2014, 2015; Supplementary Table S3: Clinical associations with serial visceral adipose tissue (VHI)^ measurements over 24 months; Supplementary Table S4: Clinical associations with serial fat mass index (FMI) measurements over 24 months; Supplementary Table S5: Clinical associations with serial appendicular skeletal muscle index (ASMI) measurements over 24 months; Supplementary Table S6: Clinical associations with serial bone mineral density measurements (lumbar spine *t*-score) over 24 months.
