# Supplementary material for: Obesity in Inflammatory Bowel Disease: Gains in Adiposity despite High Prevalence of Myopenia and Osteopenia
Source: Nutrients. 2018 Sep 1;10(9):1192. doi: 10.3390/nu10091192 (PMC6163971; doi:10.3390/nu10091192)
Supplement: Supplementary file 1 [file nutrients-10-01192-s001.pdf]

nutrients

MDPI

Supplementary Materials

Supplementary Table 1: Baseline clinical and nutritional characteristics of IBD Cohort

		Overall	Crohn's disease		Ulcerative colitis
**Patients (n)**		154	108 (70%)		46 (30%)
**Ethnicity**	Caucasian	145 (94%)	105 (97%)		40 (87%)
	Asian	6 (4%)	2 (2%)		4 (9%)
	Black	3 (2%)	1 (1%)		2 (4%)
**Male (n, %)**		85 (55%)	56 (52%)		29 (63%)
**Age (years) (median, IQR)**		31 (25-40)	31 (27-41)		31 (23-40)
**Smoking**	Current	32 (21%)	29 (27%)		2 (4%)
	Ex-smoker	39 (25%)	29 (27%)		10 (22%)
	Never smoked	83 (54%)	50 (46%)		33 (72%)
**Alcohol use (>20g ethanol/day)**		6 (4%)	4 (3%)		2 (1%)
**Age at IBD diagnosis (median, IQR)**		22 (17-29)	21 (17-27)		24 (17-30)
**Montreal criteria**		A1 31 (20%)	A1 20 (19%)		A1 11 (24%)
		A2 116 (75%)	A2 82 (76%)		A2 34 (74%)
		A3 7 (5%)	A3 6 (5%)		A3 1 (2%)
**IBD disease duration (months)**	Mean ± SD	113 ± 88	122.2 ± 90		92 ± 79
	Median, IQR	92 (54-148)	101 (59-150)		72 (42- 119)
**IBD phenotype**			L1 33 (31%)	B1 50 (46%)	E1 2 (4%)
**Montreal criteria**			L2 36 (33%)	B2 40 (37%)	E2 13 (28%)
			L3 39 (36%)	B3 18 (14%)	E3 31 (67%)
**Extra-intestinal manifestations**	Overall	40 (26%)	26 (24%)		14 (30%)
	PSC	9 (6%)	5 (5%)		4 (9%)
	Arthropathy	17 (11%)	12 (11%)		5 (11%)
	Skin lesion	8 (5%)	6 (6%)		2 (4%)
	Other	6 (4%)	3 (3%)		3 (7%)

**IBD-related surgery**		- Overall 44 (41%)<br>- Ileal/small bowel resection 30 (28%)<br>- Ileocolonic resection 10 (9%)<br>- Colectomy 4 (4%)<br>- Multiple prior surgeries 11 (10%)	- Overall 1 (2%)<br>Colectomy 1 (2%)
**IBD Clinical disease activity score** Mean ± SD		CDAI 95 ± 98	Partial Mayo 1.8 ± 2.6
Median, IQR		68 (26-138)	0 (0-4)
**C-reactive protein (mg/L)** Mean ± SD	8.42 ± 20	10 ± 23	3.6 ± 6
Median, IQR	1.95 (0.5- 8.4)	2.3 (0.5-11)	1.0 (0.3- 2.9)
**Faecal calprotectin (µg/g)** Mean ± SD	233.6 ± 269	240 ± 294	201 ± 288
Median, IQR	135 (20-273)	86 (20-430)	57 (20-220)
**Composite disease activity assessment§** (n, active disease (%))	78 (51%)	47 (44%)	19 (41%)
**Corticosteroids^** Current (n, %)	45 (29%)	31 (29%)	14 (30%)
Median, IQR	6 (1-24)	6 (0.6-24)	6 (2.7-18)
Use ≥12 months (n, %)	64 (42%)	47 (44%)	17 (37%)
**Biologic therapy (n, %)** Overall	61 (40%)	55 (51%)	6 (13%)
Infliximab	40 (26%)	36 (33%)	4 (9%)
Adalimumab	19 (12%)	18 (17%)	1 (2%)
Vedolizumab	2 (1%)	1 (1%)	1 (2%)
**5-ASA therapy (n, %)**	70 (45%)	35 (32%)	35 (76%)
**Immunomodulator (n, %)** Overall	86 (56%)	65 (60%)	21 (46%)
Azathioprine	60 (39%)	45 (42%)	15 (33%)
Mercaptopurine	6 (4%)	5 (5%)	1 (2%)
Methotrexate	3 (2%)	2 (2%)	1 (2%)
Thiopurine/allopurinol	17 (11%)	13 (9%)	4 (9%)
**International Physical Activity Questionnaire¶**			
Continuous Mean ± SD	4310 ± 5895	4408 ± 6259	4997 ± 5194
Median, IQR	2160 (693- 5664)	1671 (816- 5163)	3144 (1451- 7461)

Categorical	Low	64 (42%)	49 (45%)	15 (33%)
	Medium	38 (25%)	24 (22%)	14 (30%)
	High	32 (21%)	22 (20%)	10 (22%)
**Albumin (g/dL)**	Mean ±SD	40 ± 5	39 ± 4	40 ± 11
	Median, IQR	40 (37-43)	40 (36-43)	41 (38-44)
**Haemoglobin (g/L)**	Mean ±SD	140 ± 15	140 ± 16	139 ± 24
	Median, IQR	141 (131-150)	140 (129-150)	143 (133- 150)
**Ferritin (ng/ml)**	Mean ±SD	87 ± 82	83 ± 88	94 ± 69
	Median, IQR	63 (34-106)	56 (30-100)	85 (35- 136)
	Iron deficient	17 (11%)	13 (12%)	4 (9%)
**Calcium (mmol/L)**	Mean ±SD	2.36 ± 0.11	2.36 ± 0.11	2.36 ± 0.10
	Median, IQR	2.36 (2.29- 2.43)	2.36 (2.29- 2.42)	2.37 (2.29-2.43)
**Vitamin D nmol/ml**	Mean ±SD	67 ± 40	64 ± 28	75 ± 57
	Median, IQR	63 (43-84)	63 (42-84)	63 (51-84)
	Vitamin D deficient# n, (%)	61 (40%)	44 (41%)	17 (37%)
	Vitamin D replacement n (%)	59 (38%)	43 (40%)	16 (36%)
**Quality of life (Short Inflammatory Bowel Disease Questionnaire)**				
	Mean ± SD	50 ± 12	49 ± 13	51 ± 11
	Median, IQR	52 (43-59)	52 (41- 60)	53 (46-59)

Table legend: *Data presented as mean ± standard deviation (SD), median (interquartile range (IQR)), counts and percentage. CDAI, Crohn's Disease Activity Index; §Composite disease activity assessment using clinical indices (CDAI or Partial Mayo) and biomarker of inflammation (faecal calprotectin and C-reactive protein). ^Cumulative months equivalent to prednisolone ≥10mg daily. ¶International Physical Activity Questionnaire (Short). #Low Vitamin D level classified as <50 nmol/L, vitamin D supplementation (≥1000 IU/day).*

Supplementary Table 2: Body mass index and waist circumference by age as compared to data from the Australian Bureau of Statistic National Health Survey First Results 2014,2015

	Australian Health Survey 2014-2015			IBD Cohort (24 months)			
**Body mass index**							
	**Overall (mean)**	**Obese (%)**	**Overweight/ obese (%)**	**Patient number**	**Overall (mean ± SD)**	**Obese (%)**	**Overweight/ obese (%)**
Age 18-25	25.2	17.1%	38.9%	n = 17	24.8 ± 5.5	17.6%	41.2%
Male	25.5	17.3%	43.8%	n= 10	25.9 ± 6.5	30%	50%
Female	24.8	17.3%	33.3%	n= 7	23.1 ± 3.5	0%	28.6%
Age 25-35	26.2	19.0%	52.4%	n= 38	26.7 ± 4.1	21.1%	55.3%
Male	26.9	20.8%	62.5%	n= 25	26.4 ± 3.7	20%	56%
Female	25.5	17.3%	42.5%	n=13	27.3 ± 4.9	23.1%	53.9%
Age 35-45	27.6	28.6%	65.9%	n= 35	28.8 ± 6.5	34.3%	68.6%
Male	27.7	26.7%	74.3%	n= 19	27.5 ± 5.9	21.1%	63.2%
Female	27.6	30.7%	58.1%	n= 16	30.5 ± 7.1	50%	75%
Age 45-55	28.3	33%	70.6%	n= 20	30.3 ± 5.4	55%	80%
Male	28.6	33.2%	79.8%	n= 12	31.6 ± 4.7	58.3%	91.7%
Female	27.9	33%	61.9%	n=8	28.3 ± 6.1	50.0%	62.5%
**Waist circumference**							
	**Overall (mean)**	**At risk waist circumference**		**Patient number**	**Overall (mean ± SD)**	**At risk waist circumference**	
Age 18-25				n = 17	88.0 ± 14.6		
Male	88	27.3%		n= 10	93.4 ± 14.9	50%	
Female	79.4	34.5%		n= 7	80.2 ± 11.1	42.9%	
Age 25-35				n= 38	91.0 ± 11.7		
Male	92.9	41.7%		n= 25	93.3 ± 11.9	40%	
Female	82.3	49.1%		n=13	86.6 ± 10.4	69.2%	
Age 35-45				n= 35	97.0 ± 15.4		
Male	96.5	55.7%		n= 19	98.1 ± 15.7	47.4%	

Female	87.5	66.9%	n= 16	95.7 ± 15.5	75%
Age 45-55<br>Male<br>Female	 100.1<br>89.1	 69.8%<br>71.2%	n= 20<br>n= 12<br>n=8	100.3 ± 14.27<br>105.7 ± 9.97<br>92.13 ± 16.45	 91.7%<br>87.5%

Table legend: *IBD cohort body composition data derived from 24-month dataset. Data presented as mean ± standard deviation, median (interquartile range), counts and percentage. SD, standard deviation. Comparative data derived from the Australian Bureau of Statistics National Health Survey 2014-2015 URL:http://www.abs.gov.au/ausstats/abs@.nsf/Lookup/by%20Subject/4364.0.55.001~2014-15~Main%20Features~Key%20findings~1. Overweight and obese characterisation according to WHO criteria. At risk waist circumference defined as >80cm for women and >94cm for men.*

Supplementary Table 3: Clinical associations with serial visceral adipose tissue (VHI)^ measurements over 24 months

Variable		Univariable		Full multivariable model	
		Est. (95% CI)	P value	Est. (95% CI)	P value
Time	Repeated measures over 24m	0.07 [0.02, 0.13]	0.006	-0.015 [-0.072, 0.043]	0.61
Demographics	Age at study entry	0.062 [0.044, 0.080]	<0.0001	0.040 [0.023, 0.057]	<0.0001
	Gender (Male vs. female)	0.7 [0.3, 1.1]	0.0009	0.67 [0.39, 0.95]	<0.0001
IBD-related factors	IBD phenotype (Ulcerative colitis vs. Crohn's disease)	-0.26 [-0.72, 0.20]	0.25	-0.19 [-0.47, 0.09]	0.16
	IBD disease duration	0.003 [0.0011, 0.005]	0.002	-0.0007 [-0.0022, 0.0009]	0.36
	Faecal calprotectin (μg/g)	-0.00016 [-0.00042, 0.0001]	0.2	0.0000 [-0.00027, 0.00026]	0.97
	C- reactive protein (mg/L)	-0.0009 [-0.0054, 0.0036]	0.69	0.0007 [-0.0038, 0.0052]	0.75
	Steroid use	-0.03 [-0.18, 0.12]	0.65	0.05 [-0.10, 0.20]	0.50
	Biologic therapy	0.06 [-0.06, 0.19]	0.31	0.00 [-0.11, 0.11]	0.95
	Immunomodulator therapy	0.07 [-0.03, 0.17]	0.17	0.011 [-0.064, 0.086]	0.78
Lifestyle and nutritional factors	Smoking status<br>Current vs. Never<br>Ex vs. Never	 0.50 [0.0, 1.0]<br>0.50 [0.0, 1.0]	0.04	 -0.08 [-0.43, 0.28]<br>-0.03 [-0.34, 0.28]	0.91
	Excess alcohol intake¶	0.6 [-0.4, 1.7]	0.23	0.20 [-0.44, 0.84]	0.51
	Vitamin D level (nmol/ml)	-0.0019 [-0.0045, 0.0008]	0.17	-0.0022 [-0.0046, -0.0002]	0.06
	Habitual exercise (IPAQ score, continuous)§	0.001 [-0.008, 0.010]	0.81	-0.0008 [-0.0089, 0.0074]	0.82

	Albumin (g/dL)	-0.002 [-0.019, 0.016]	0.86	-0.004 [-0.021, 0.013]	0.66
Body composition factors	Body mass index (BMI)	0.12 [0.10, 0.14]	<0.0001	0.08 [0.06, 0.11]	<0.0001*
	Waist circumference	0.035 [0.029, 0.042]	<0.0001	0.015 [0.005, 0.026]	0.003*
	Waist:hip ratio	1.1 [0.4, 1.7]	0.0009	-0.2 [-1.0, 0.6]	0.54
	Grip strength (pounds per square inch, PSI)	0.001 [-0.009, 0.011]	0.84	-0.007 [-0.016, 0.002]	0.13

Table legend: *^VHI, visceral adipose tissue area (cm^3)/ height (m)^2, log-transformed prior to analysis; ¶Defined according to Australian healthy Drinking guidelines; §International Physical Activity Questionnaire for assessment of habitual physical activity. Linear mixed effects regression models with missing data imputed with cohort means.*

Supplementary Table 4: Clinical associations with serial fat mass index (FMI) measurements over 24 months

Variable		Univariable		Full multivariable model	
		Est. (95% CI)	P value	Est. (95% CI)	P value
Time	Repeated measures over 24m	0.34 [0.18, 0.50]	<0.0001	0.04 [-0.13, 0.20]	0.65
Demographics	Age at study entry	0.18 [0.11, 0.25]	<0.0001	0.024 [-0.010, 0.058]	0.17
	Gender (Male vs. female)	-2.7 [-4.2, -1.2]	0.0004	-2.2 [-2.8, -1.6]	<0.0001
IBD-related factors	IBD phenotype (Ulcerative colitis vs. Crohn's disease)	-0.7 [-2.5, 1.0]	0.41	-0.16 [-0.73, 0.41]	0.56
	IBD disease duration	0.011 [0.004, 0.019]	0.002	-0.0009 [-0.0040, 0.0021]	0.53
	Faecal calprotectin (µg/g)	-0.0009 [-0.0017, -0.0001]	0.02	0.00007 [-0.00063, 0.00078]	0.82
	C- reactive protein (mg/L)	-0.007 [-0.019, 0.005]	0.24	-0.001 [-0.011, 0.010]	0.93
	Steroid use	-0.11 [-0.58, 0.36]	0.64	0.34 [-0.08, 0.75]	0.09
	Biologic therapy	0.31 [-0.11, 0.73]	0.14	0.14 [-0.12, 0.41]	0.26
	Immunomodulator therapy	0.24 [-0.11, 0.58]	0.17	-0.09 [-0.25, 0.08]	0.24
Lifestyle and nutritional factors	Smoking status<br>Current vs. Never<br>Ex vs. Never	 0.5 [-1.6,2.6]<br>0.8 [-1.0, 2.7]	0.65	 -0.28 [-0.98, 0.43]<br>-0.7 [-1.3, -0.1]	0.07
	Excess alcohol intake^	-0.6 [-4.7, 3.4]	0.75	0.0 [-1.3, 1.3]	0.97
	Vitamin D level (nmol/ml)	-0.0003 [-0.0084, 0.0079]	0.95	-0.007 [-0.013, -0.001]	0.02
	Habitual exercise (IPAQ score, continuous)§	-0.011 [-0.039, 0.018]	0.46	-0.020 [-0.042, 0.003]	0.07
	Albumin (g/dL)	0.029 [-0.027, 0.085]	0.30	-0.002 [-0.049, 0.044]	0.91

Body composition factors	Body mass index (BMI)	0.57 [0.52, 0.62]	<0.0001	0.52 [0.45, 0.59]	<0.0001*
	Waist circumference	0.12 [0.10, 0.14]	<0.0001	0.07 [0.04, 0.10]	<0.0001*
	Waist:hip ratio (WHR)	2.6 [0.6, 4.5]	0.008	-3.0 [-5.2, -0.8]	0.006*
	Grip strength (pounds per square inch, PSI)	-0.033 [-0.068, 0.001]	0.06	-0.044 [-0.066, -0.021]	<0.0001*

Table legend. *^Excess alcohol use defined according to Australian healthy Drinking guidelines; §IPAQ, International Physical Active Questionnaire; FMI, fat mass index (kg/ height m²); ASMI, appendicular skeletal muscle index (kg/ height m²); Linear mixed effects regression models with missing data imputed with cohort means.*

Supplementary Table 5: Clinical associations with serial appendicular skeletal muscle index (ASMI) measurements over 24 months

**Variable**		**Univariable**		**Full multivariable model**	
		Est. (95% CI)	P value	Est. (95% CI)	P value
Time	Repeated measures over 24m	-0.06 [-0.11, -0.02]	0.006	-0.16 [-0.21, -0.10]	<0.0001*
Demographics	Age at study entry	0.010 [-0.013, 0.033]	0.40	0.005 [-0.010, 0.021]	0.53
	Gender (Male vs. female)	1.5 [1.0, 1.9]	<0.0001	1.2 [0.9, 1.4]	<0.0001*
IBD-related factors	IBD phenotype (Ulcerative colitis vs. Crohn's disease)	0.07 [-0.49, 0.62]	0.81	0.01 [-0.25, 0.28]	0.93
	IBD disease duration	-0.0009 [-0.0031, 0.0013]	0.43	0.0003 [-0.0011, 0.0018]	0.61
	Faecal calprotectin (µg/g)	-0.00047 [-0.00070, -0.00025]	<0.0001	-0.00043 [-0.00067, -0.00018]	0.0004*
	C- reactive protein (mg/L)	-0.0020 [-0.0055, 0.0015]	0.25	-0.0001 [-0.0037, 0.0035]	0.95
	Steroid use	-0.05 [-0.19, 0.08]	0.43	-0.05 [-0.19, 0.09]	0.50
	Biologic therapy	-0.06 [-0.18, 0.07]	0.37	-0.04 [-0.15, 0.06]	0.37
	Immunomodulator therapy	0.08 [-0.02, 0.18]	0.11	0.07 [0.00, 0.14]	0.05
Lifestyle and nutritional factors	Smoking status<br>Current vs. Never<br>Ex vs. Never	 0.10 [-0.55, 0.75]<br>0.5 [-0.1, 1.1]	 0.22	 -0.06 [-0.39, 0.27]<br>0.19 [-0.16, 0.40]	 0.50
	Excess alcohol intake^	-0.2 [-1.5, 1.0]	0.69	-0.7 [-1.3, -0.1]	0.02*
	Vitamin D level (nmol/ml)	-0.0004 [-0.0027, 0.0020]	0.75	0.0008 [-0.0014, 0.0031]	0.43
	Habitual exercise (IPAQ score, continuous)§	0.003 [-0.005, 0.012]	0.44	0.004 [-0.004, 0.012]	0.32

	Albumin (g/dL)	0.017 [0.000, 0.033]	0.04	-0.001 [-0.017, 0.016]	0.96
Body composition factors	Body mass index (BMI)	0.10 [0.08, 0.12]	<0.0001	0.13 [0.11, 0.15]	<0.0001*
	Grip strength (pounds per square inch, PSI)	0.019 [0.009, 0.030]	0.0007	0.020 [0.011, 0.028]	<0.0001*

Table legend: *^Excess alcohol use defined according to Australian healthy Drinking guidelines; §IPAQ, International Physical Active Questionnaire; FMI, fat mass index (kg/ height m^2); ASMI, appendicular skeletal muscle index (kg/ height m^2); Linear mixed effects regression models with missing data imputed with cohort means.*

Supplementary Table 6: Clinical associations with serial bone mineral density measurements (lumbar spine *t*- score) over 24 months

Variable		Univariable		Full multivariable model	
		Est. (95% CI)	P value	Est. (95% CI)	P value
Time	Repeated measures over 24m	0.012 [-0.015, 0.040]	0.38	0.06 [0.01, 0.10]	0.01*
Demographics	Age at study entry	-0.008 [-0.025, 0.010]	0.39	-0.006 [-0.034, 0.023]	0.68
	Gender (Male vs. female)	-0.34 [-0.75, 0.07]	0.10	-0.38 [-0.82, 0.07]	0.08
IBD-related factors	IBD phenotype (ulcerative colitis vs. Crohn's disease)	-0.17 [-0.64, 0.29]	0.46	-0.26 [-0.74, 0.21]	0.26
	IBD disease duration	-0.0012 [-0.0028, 0.0004]	0.15	-0.0031 [-0.0057, -0.0005]	0.01*
	Faecal calprotectin (µg/g)	0.00002 [-0.00013, 0.00016]	0.83	0.00004 [-0.00013, 0.00021]	0.61
	C- reactive protein (mg/L)	-0.0019 [-0.0040, 0.0001]	0.06	-0.0019 [-0.0042, 0.0004]	0.08
IBD therapy	Steroid use	0.07 [-0.01, 0.15]	0.07	0.10 [0.01, 0.18]	0.02*
	Biologic therapy	0.07 [0.00, 0.14]	0.04	0.08 [0.01, 0.16]	0.02*
	Immunomodulator therapy	0.013 [-0.054, 0.080]	0.69	-0.022 [-0.093, 0.050]	0.53
Lifestyle and nutritional factors	Smoking status				
	Current vs. Never	-0.33 [-0.87, 0.21]	0.35	-0.23 [-0.83, 0.37]	0.61
	Ex vs. Never	0.09 [-0.40, 0.57]		0.06 [-0.45, 0.57]	
	Excess alcohol intake^	-0.9 [-1.9, 0.1]	0.07	-0.2 [-1.3, 0.8]	0.65
	Vitamin D level (nmol/ml)	-0.0004 [-0.0018, 0.0009]	0.51	-0.0003 [-0.0017, 0.0011]	0.70
	Habitual exercise (IPAQ score, continuous)§	0.0003 [-0.0044, 0.0051]	0.88	0.0000 [-0.0048, 0.0048]	1.00

	Calcium	-0.22 [-0.58, 0.14]	0.23	-0.30 [-0.71, 0.10]	0.12
	Albumin (g/dL)	0.002 [-0.008, 0.011]	0.70	0.006 [-0.005, 0.018]	0.25
Body composition factors	BMI	0.004 [-0.009, 0.017]	0.51	0.007 [-0.011, 0.025]	0.42
	Grip strength (pounds per square inch, PSI)	0.006 [0.000, 0.012]	0.06	0.005 [-0.001, 0.012]	0.10
	Fat mass index (FMI)	0.007 [-0.012, 0.026]	0.44	-0.006 [-0.034, 0.021]	0.65
	Appendicular skeletal muscle index (ASMI)	-0.003 [-0.067, 0.061]	0.93	0.005 [-0.073, 0.083]	0.89
	Functional sarcopenia¶	-0.07 [-0.20, 0.06]	0.29	-0.09 [-0.23, 0.05]	0.19

Table legend: *^Excess alcohol use defined according to Australian healthy Drinking guidelines; §IPAQ, International Physical Active Questionnaire; FMI, fat mass index (kg/ height m^2); ASMI, appendicular skeletal muscle index (kg/ height m^2); ¶functional sarcopenia, low ASMI and grip strength ≥1 standard deviation below mean. *Significant P value (<0.05;) Linear mixed effects regression models with missing data imputed with cohort means.*
